# Emergency Open Incarcerated Hernia Repair with a Biological Mesh in a Patient with Colorectal Liver Metastasis Receiving Chemotherapy and Bevacizumab Uncomplicated Wound Healing

**DOI:** 10.1155/2014/848030

**Published:** 2014-12-21

**Authors:** Alexandros Giakoustidis, Dawn Morrison, Kyriakos Neofytou, Dimitrios Giakoustidis, Satvinder Mudan

**Affiliations:** ^1^Department of Surgery, The London Clinic, 20 Devonshire Pl, London W1G 6BW, UK; ^2^Upper GI/HPB Unit, Department of Academic Surgery, Royal Marsden Hospital, Fulham Roadd, London SW3 6JJ, UK; ^3^Division of Transplantation, Department of Surgery, Hippokration General Hospital, Aristotle University of Thessaloniki, 49 Kostantinoupoleos Street, 54642 Thessaloniki, Greece

## Abstract

Bevacizumab is a humanized monoclonal antibody targeting vascular endothelial growth factor (VEGF), often used in combinational chemotherapy regimens for the treatment of patients with colorectal liver metastases. However adverse events have been attributed to the use of bevacizumab including gastrointestinal perforations, thrombotic events, hypertension, bleeding, and wound healing complications. 53-year-old male, with a history of colorectal cancer with liver metastasis, receiving a combination of cytotoxic chemotherapy (FOLFIRI, irinotecan with fluorouracil and folinic acid) with bevacizumab presented as an emergency with an incarcerated incisional hernia. The last administration of chemotherapy and bevacizumab had taken place 2 weeks prior to this presentation. As the risk of strangulation of the bowel was increased, a decision was made to take the patient to theatre, although the hazard with respect to wound healing, haemorrhage, and infection risk was high due to the recent administration of chemotherapy with bevacizumab. The patient underwent an open repair of the incarcerated recurrent incisional hernia with placement of a biological mesh, and the postoperative recovery was uncomplicated with no wound healing or bleeding problems.

## 1. Introduction

It has been reported that incisional hernias are a common complication following abdominal surgery with an incidence ranging between 3 and 13% [1]. Wound infection and hernia recurrence are among the most common complications of any ventral and incisional hernia repair [2, 3].

Bevacizumab is an antiangiogenic agent and specifically a humanized monoclonal antibody which targets the vascular endothelial growth factor (VEGF), resulting in the attenuation of neoangiogenesis, which is essential for the further development of malignancies. The addition of bevacizumab to chemotherapy regimens has been reported to increase the response of liver metastases when compared to conventional chemotherapies [4, 5].

However there has been raised concern regarding bevacizumab implications on hepatocyte proliferation, hepatic recovery, and wound healing [6, 7]. Surgery followed by its administration has been linked with other possible complications as well such as gastrointestinal perforations, thrombotic events, hypertension, and bleeding. We present a case of a patient who underwent an emergency open incarcerated hernia repair with a biological mesh on the background of bevacizumab administration for colorectal liver metastases.

## 2. Case Report

A 53-year-old male, with a history of colorectal cancer with liver metastasis, was diagnosed in March 2009. He had colon resection at that time, with histopathology showing T4N2M1 and 10/18 lymph nodes involved. He had liver resection following 4 cycles of FOLFOX (oxaliplatin with fluorouracil and folinic acid) and since then he was administered a combination of chemotherapy (FOLFIRI) with bevacizumab for management of the progression of the disease to the peritoneum and subcutaneous nodules. Parallel to this, he had developed an incisional hernia from his abdominal wound, with a previous attempt to repair it by his primary surgeon, resulting however in a recurrent incisional hernia.

**Figure 1:**
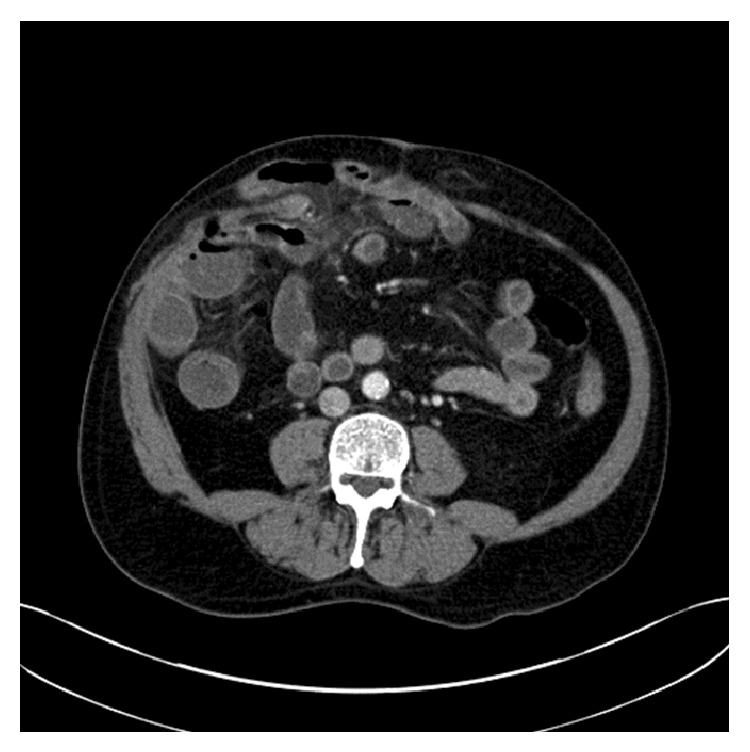
A CT image demonstrating the recurrent incisional hernia containing bowel loops.

He then presented as an emergency with an incarcerated recurrent incisional hernia with increased abdominal pain. A CT scan performed on the day of presentation demonstrated abdominal hernias containing loops of small bowel without evidence of obstruction (Figure 1). The last administration of FOLFIRI and bevacizumab had taken place 2 weeks prior to his presentation. As the risk of strangulation of the bowel was increased, a decision was made to take the patient to theatre, although the hazard with respect to wound healing, haemorrhage, and infection risk was high due to the recent administration of chemotherapy with bevacizumab. The patient underwent an open repair of the incarcerated recurrent incisional hernia with placement of a biological mesh, and the postoperative recovery was uncomplicated with no wound healing or bleeding problems. At the last follow-up, 1 year from incisional hernia repair, there were no evidences of hernia recurrence.

## 3. Discussion

In recent years the monoclonal antibody bevacizumab, which inhibits angiogenesis by binding to the VEGF, has been used more often in chemotherapy regimens for patients with CRLM [8].

However the use of bevacizumab has raised concerns as there have been adverse events linked with its administration including gastrointestinal perforations, haematologic toxicity, thrombotic events, hypertension, proteinuria, bleeding, and wound healing complications [9–12]. Most surgical teams would wait 6–8 weeks before they proceed to hepatectomy after neoadjuvant administration of bevacizumab in patients with colorectal liver metastases, with this tactic being outlined because of the relatively long half-life of bevacizumab, which is 21 days. However it has been reported that surgery sooner than 8 weeks following discontinuation of bevacizumab leads to increased complications, although there is a study data supporting the fact that this time interval does not actually affect the percentage of perioperative complications [13].

On the other hand incisional hernias can occur as a complication in up to 20% of abdominal operations [14]. A great technical challenge is involved when an incarcerated incisional hernia with associated bowel obstruction in contaminated field is repaired as an emergency [15]. Additionally it has been reported that there can be high rates of wound infection in repairs of incarcerated incisional hernias and associated bowel obstruction, with prosthetic mesh [16]. In an experimental study from Spain, the host tissue's incorporation of collagen bioprostheses and a synthetic absorbable prosthesis was assessed [17]. Pascual et al. reported that the tissue infiltration of laminar absorbable prostheses can be affected by the mesh's structure and composition. Additionally Pascual et al. found that the synthetic prosthesis exhibited a distinct pattern of tissue incorporation and a higher macrophage response when compared to the biological prostheses, concluding that, between the laminar, absorbable biomaterials that they tested, the same biological mesh that we used demonstrated better levels of integration and degradation [17].

In this case our patient had undergone colon and liver resection for a primary colon tumour with liver metastases and was receiving cytotoxic chemotherapy with bevacizumab for progressive disease. This was complicated by the occurrence of a huge incarcerated recurrent incisional hernia repair which required immediate intervention. An open incisional hernia repair was carried out as an emergency with placement of a biological mesh, with the last administration of bevacizumab 2 weeks prior to the operation date. However in spite of all the relative risks mentioned before, there was an uncomplicated postoperative recovery without any wound healing problems, bleeding, or presence of collections.

## 4. Conclusion

Bevacizumab administration in a patient undergoing emergency operation did not have an impact on the postoperative recovery and wound healing.
